# The Pyroptosis-Related Risk Genes APOBEC3D, TNFRSF14, and RAC2 Were Used to Evaluate Prognosis and as Tumor Suppressor Genes in Breast Cancer

**DOI:** 10.1155/2022/3625790

**Published:** 2022-08-25

**Authors:** Qian Chen, He Jun, ChengGuang Yang, Feng Yang, YingJie Xu

**Affiliations:** ^1^General Surgery, Tongren Hospital, Shanghai Jiaotong University School of Medicine, Shanghai, China; ^2^General Surgery, The Third Affiliated Hospital of Naval Military Medical University, Shanghai, China

## Abstract

**Background:**

Pyroptosis is a type of cell death that plays an important role in predicting prognosis and immunoregulation in cancers. However, the pyroptosis-related gene signature for prognosis and immune infiltration prediction has not been studied in breast cancer (BC).

**Methods:**

The Gene Expression Omnibus (GEO) and Cancer Genome Atlas (TCGA) databases were used to obtain the expression and clinical data of genes. 52 pyroptosis-related genes were obtained from TCGA-BC and estimated differentially expressed genes by the limma program. To categorize the molecular subtypes of pyroptosis-related genes, the ConsensusClusterPlus tool was utilized. Cox and Lasso regression analyses were used to create a signature. TCGA-BC dataset as the training set and the GSE37751 test set for risk research. Gene set enrichment analysis (GSEA) was used to conduct KEGG and GO studies of subtype groups. We also used the ssGSEA approach in the GSVA package to calculate the risk score of immune cells. Finally, pyroptosis-related genes in BC were validated using qPCR and immunohistochemical assays. Clone formation and EDU assays were used to explore the ability of signature genes to regulate the proliferation of BC cells.

**Results:**

Based on pyroptosis-related genes, the C1 and C2 subtypes were obtained. Survival analysis results showed that the C2 group had a better prognosis. Then, a three-gene signature (APOBEC3D, TNFRSF14, and RAC2) were created by Lasso regression analysis, which had a good prediction effect in the TCGA-BC and GSE37751 datasets. Our nomogram has a fair degree of accuracy in predicting the survival rates of BC patients. The pyroptosis-related signature has a good predictive effect in evaluating the tumour microenvironment score, 28 types of immune cells and response to immune checkpoint therapy. Finally, qPCR and immunohistochemistry staining results indicated that APOBEC3D, TNFRSF14, and RAC2 expression in BC tissues was low. The results of clone formation and EdU assays showed that high expression of signature genes inhibited the proliferation ability of BC cells.

**Conclusions:**

Based on pyroptosis-related genes (APOBEC3D, TNFRSF14, and RAC2), we built a novel prognostic molecular model for BC that might be used to assess prognostic risk and immune infiltration in BC patients. These signature genes are also tumor suppressor genes and may serve as potential targets for BC.

## 1. Introduction

Breast cancer (BC) is the main health concern and is the most prevalent tumour among females worldwide. It is estimated that in 2020, 4.57 million new BC cases will be detected, and approximately 680,000 people are expected to die from BC [1]. The NCCN guidelines recommend that BC is mainly treated with surgery, chemotherapy and antioestrogen therapy [2]. However, the value of treatment is not very good among advanced BC patients. BC is a diverse tumor with four major molecular subgroups; therefore, finding new biomarkers is still vital for early diagnosis and treatment methods. Pyroptosis is the process of gasdermin-mediated programmed cell death (PCD), which is known to involve extracellular responses and has been widely studied in many cancers [3]. Pyroptosis has been shown to successfully remove malignant cells and provide novel cancer treatment strategies [4]. Surprisingly, inflammasome-mediated pyroptosis has been linked to tumor formation and immunology in recent research [5]. As a result, finding a pyroptosis-related signature to predict BC prognosis and treatment methods is extremely important.

The TCGA project, which provides a comprehensive genetic examination of various malignancies and demonstrates links with clinical outcomes. In addition, the tumour project of TCGA includes mutations, genomic copy number changes, transcriptome, and methylation profiles [6]. To characterize molecular profiles, researchers combined information from transcriptome RNA sequencing with applied genomic characterizations, which revealed potential druggable targets for female tumors including BC [7–9]. In addition to identifying nearly all genes previously linked to BC, the researchers discovered numerous new and severely altered genes, including BRCA1 and BRCA2, which could be used as therapeutic targets.

In this study, we identified pyroptosis-related genes from TCGA-BC and used them to construct a novel predictive molecular model for BC. In addition, the model has the potential to be a useful tool for assessing prognostic risk and immune infiltration in BC patients. In conclusion, our findings imply that the signature might be utilized to assess prognosis and immune infiltration in BC and that the signature genes could be employed as possible targets for the disease.

## 2. Methods

### 2.1. Downloading Data

The TCGA dataset (GDC @ https://gdc.cancer.gov/) (47 nontumor samples and 1096 tumor samples) was used to obtain expression data and clinical follow-up information for BC patients. GEO (https://www.ncbi.nlm.nih.gov/geo/) has made the GSE37751 datasets (112 nontumor samples and 61 tumor samples) publicly available. From the literature, we gathered 52 pyroptosis-related genes [3, 10–12].

### 2.2. Molecular Subtype Identification

Limma software was used to analyse the differentially expressed genes (DEGs) based on the threshold false discovery rate (FDR) < 0.05 after the 52 pyroptosis-related genes expression data were matched with the TCGA-BC dataset. Next, ConsensusClusterPlus was used to find new molecular subclasses of BC, which provides quantitative evidence for determining the number and membership of possible clusters within the TCGA dataset.

### 2.3. Multivariate Analyses and Molecular Risk Model Construction

For the TCGA-BC dataset, we used Cox regression analysis. A *p* value of 0.05 was judged survival linked based on the results of multivariate analysis. Furthermore, the R software package glmnet for lasso Cox regression was used to compress the screened genes and used to build the risk model. We also employed the TCGA-BC dataset as the study's training set and the GSE37751 test set.

### 2.4. Analysis of Immune Scores between Clusters

The immunological score among the clusters in TCGA-BC dataset was determined using the GSVA package's single-sample gene set enrichment analysis (ssGSEA) approach. We used ESTIMATE software to estimate the tumor microenvironment score for tumor purity, StromalScore, ImmuneScore, and ESTIMATEScore. Twenty-eight different types of immune cells were evaluated using the GSVA program ssgsea. The differences in immune ratings between the molecular subtypes were then compared.

Furthermore, we analysed the correlation of the molecular risk model with immune-inhibitory markers. We collected 6 immune-inhibitory markers, including CD274, PDCD1, PDCD1LG2, CTLA4, HAVCR2, and IDO1, from the published literature. Using the chi-square test, the response to immune checkpoint therapy was estimated and compared.

### 2.5. Tissue Samples

Ten BC tissues were collected and kept at 80°C. Preoperative antitumor treatments were not given to any of the patients. Informed consent papers were signed by patients. This study was approved by the Ethics Committee of Shanghai Tongren Hospital (2021-088-02).

### 2.6. RT–qPCR Analysis

Total RNA was isolated by TRIzol reagent (Invitrogen, Thermo Scientific, Shanghai, China), and RNA was reverse-transcribed into cDNA using a HiScript II 1st Strand cDNA Synthesis Kit (Invitrogen, Thermo Scientific, Shanghai, China) (Vazyme, China). ChamQ SYBR qPCR Master Mix was used to quantify qPCR analyses (Vazyme, China).

### 2.7. Immunohistochemistry

Paraffin sections of breast cancer tissue were used for immunohistochemistry. The slides were dewaxed with methanol and rehydrated with alcohol after being dried at 60°C. The slides were then submerged in 3% hydrogen peroxide overnight and labelled with antibodies. The experiment was carried out with the manufacturer's instructions. The antibodies purchased from Abcam as follows: APOBEC3D antibody (ab105869), anti-TNFRSF14 antibody (ab47677) and anti-RAC2 antibody (ab2244). The immunohistochemistry results were evaluated under a microscope at 20 × 10. The IHC findings were analysed by Image-Pro Plus 6.0 Software.

### 2.8. Cell Lines and Transfection

The human normal mammary epithelial cell line MCF10 A, and BC cell lines (MDA-MB-231 and MCF-7) were purchased from the National Collection Authenticated Cell Cultures (Shanghai, China). All cells were incubated at 37°C and 5% CO_2_ in a incubator. Transfection was carried out by Lipofectamine 3000 reagent (Invitrogen, China, No. L3000015) according to the instructions. The coding sequences of human APOBEC3D, TNFRSF14, and RAC2 were cloned into the pEZ-M03 vector.

### 2.9. Ethynyl Deoxyuridine (EdU) Assays

The experiment was carried out exactly as instructed. Cells were cultivated at a density of 10000 cells in 96-well plates per well. The 96-well plates were then incubated for 3 hours at 37°C with 10 M EdU labelling medium (Beyotime Biotechnology, Shanghai, China). After fluorescence microscopy inspection, the percentage of EdU-positive cells was determined.

### 2.10. Colony Formation Assay

A total of 1000 cells were placed in six-well plates for the colony formation test. The cells were mixed together and grown for one week in culture media containing 10% FBS. A single colony was defined as a cluster of 30 cells or less.

### 2.11. Statistical Analysis

The SPSS 13.0 statistical software program was used to analyse the data (IBM Corporation, Armonk, NY, USA). GraphPad Prism 8.0 was used to create the graphs (GraphPad Software, Inc., San Diego, CA). Statistical significance was defined as a *p* value < 0.05.

## 3. Results

### 3.1. Identification and Molecular Pyroptosis-Related Type

**Figure 1:**
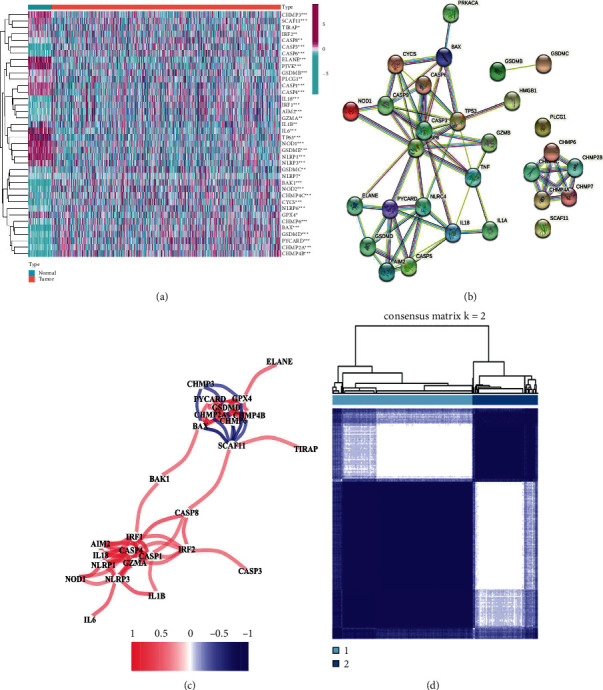
Differentially expressed pyroptosis-related genes in BC. (a). In BC, 21 genes had high expression, while 17 genes had low expression. (b). The outcome of the PPI network analysis. (c) The correlation analysis results. (d). DES clustering analysis.

**Figure 2:**
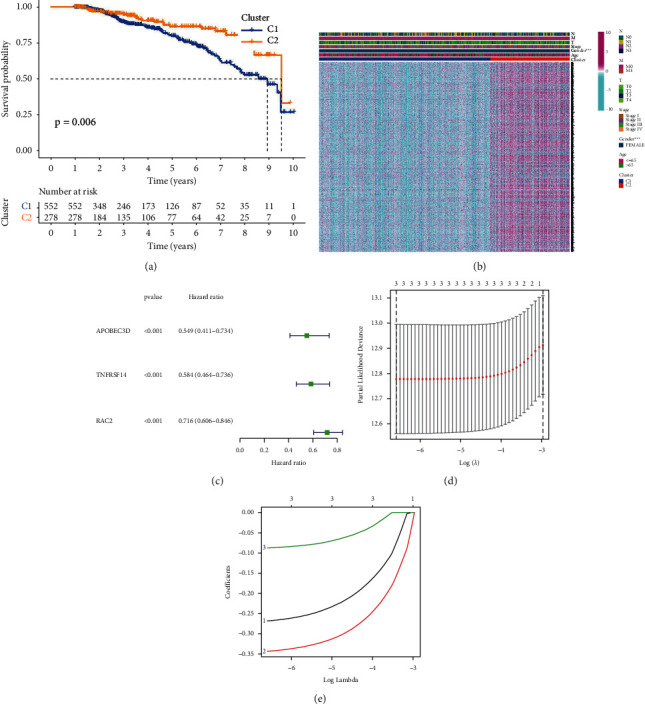
The two clusters for prognostic prediction in BC. (a). C1 had the poorest prognosis, whereas C2 had the best prognosis. (b). A total of 1190 DESs were found to be shared by the two groups. (c). APOBEC3D, TNFRSF14, and RAC2 were all found to be risk variables in a forest plot of HRs. (Both d and f). The model was built by Lasso regression.

The TCGA-BC dataset was used to calculate 52 pyroptosis-related genes expression, and 21 genes was high expression and 17 genes was low expression (Figure 1(a)) in BC. To further investigate the interrelationship among the DESs, a PPI network and correlation analysis were constructed. GSDMD and CHMP6 were shown to be linked to the risk of BC in the study (Figures 1(b) and 1(c)). The ConsensusClusterPlus tool was also used to perform clustering analysis. The 1096 BC samples were classified into C1 and C2 clusters (Figure 1(d)). As shown in Figure 2(a) (*p*=0.006), C1 had the worst prognosis, and C2 had the best prognosis in BC. In addition, we counted the differentially expressed genes based on the clusters. A total of 1190 DES (padj <0.05 and |log2FC|>1) were found to be common between the two groups (Figure 2(b)). Between the 1190 candidate DESs mentioned above and the survival data, we ran multivariate Cox regression analyses. APOBEC3D, TNFRSF14, and RAC2 were all found to be risk variables in a forest plot of HRs. To minimize the genes number for the risk model, Lasso regression was utilized (Figure 2(d)). As shown in Figure 2(e), we then utilized a 10-fold cross test to build the model and confidence interval for each lambda. The following is the final 3-gene signature formula:

RiskScore = −0.268130112241867 ∗ APOBEC3D − 0.343435308531483 ∗ TNFRSF14 − 0.0874551279062335 ∗ RAC2.

### 3.2. Risk Model Analysis and Comparison

**Figure 3:**
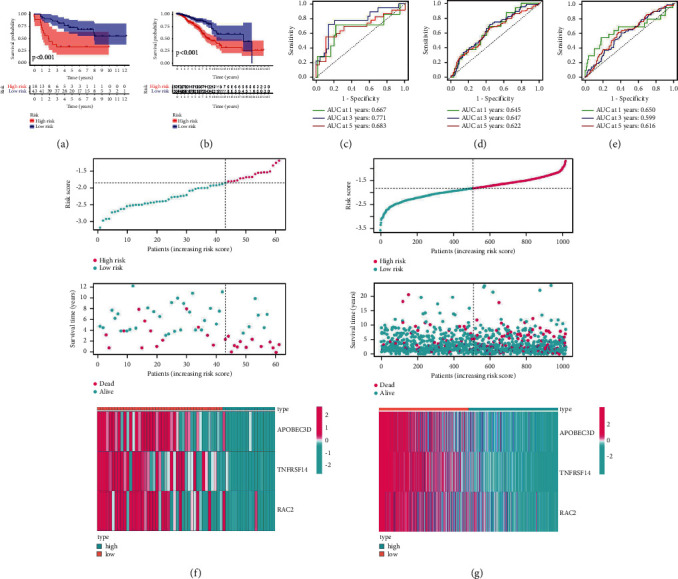
Examination of whether our signature is feasible. (a and b). In both the training and test sets, the risk model was highly connected to the prognosis of BC patients. (c and d). The prognostic classification efficiency was good according to the ROC curve data. (e). Our approach outperforms three-gene signatures in terms of accuracy. (f and g). In the risk score distribution, high-risk scores group have worse prognosis.

We used the TCGA-BC dataset as the training set and the GSE37751 test set for risk research to determine whether our signature was feasible. To validate the prognostic relevance of the risk score, the Kaplan–Meier survival curves, ROC curves, and risk score distributions for OS prediction were examined. In both the training and test sets, the risk model was highly connected to the prognosis of BC patients, as shown in Figures 3(a) and 3(b). ROC curve results showed that the prognostic prediction for 1, 3, and 5 years had good classification efficiency (Figures 3(c) and 3(d)). Three prognostic risk models (PMID 34589498) were chosen for comparison with our risk model. The 1-, 3-, and 5-year AUC values for the 3-gene signature model were lower than those for our model. This finding demonstrates that our model produces better results (Figure 3(e)). In the TCGA-BC dataset, as the risk score increased, the expression levels of APOBEC3D, TNFRSF14 or RAC2 were downregulated, and the number of surviving patients decreased (Figure 3(f)). These findings in the GSE37751 external test set, which were from different data sources, indicate that the risk signature performs well in predicting the survival of BC (Figure 3(g)).

### 3.3. Cox Regression Analysis and Nomogram Construction

**Figure 4:**
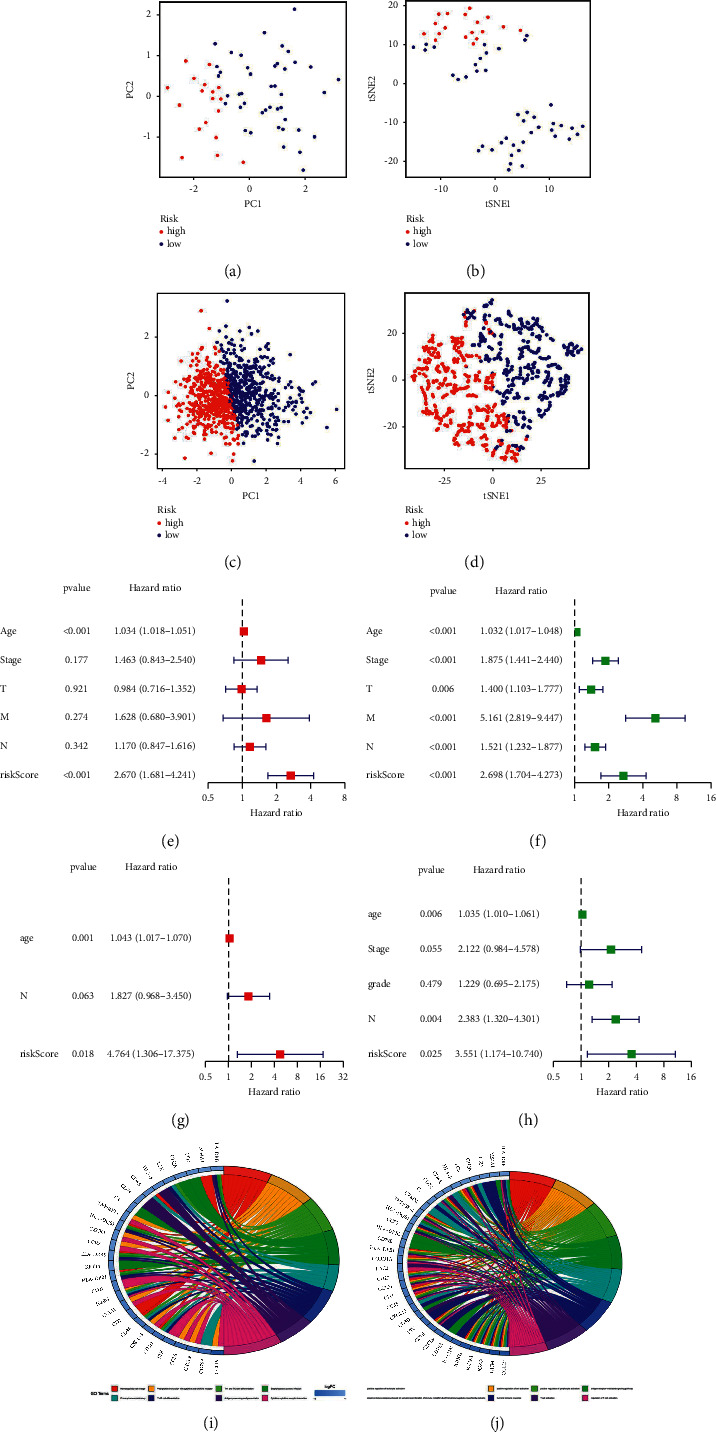
Clinical traits can be distinguished using risk models. Both the training (a and b) and test sets, risk models can accurately identify high-risk patients (c and d). In the training (e and g) and test sets, the risk model is the influence of survival time independent factors (f and h). DEGs involved in pyroptosis: (i) GO analysis and (j) KEGG pathway enrichment analysis.

**Figure 5:**
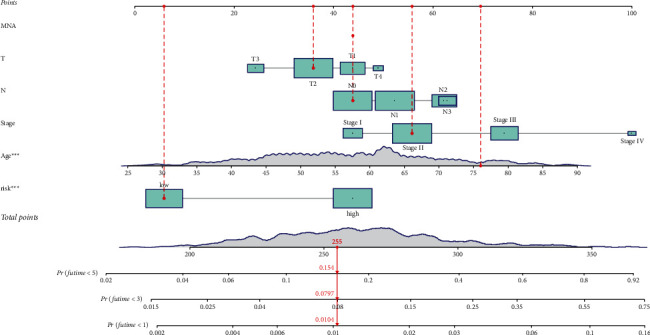
The nomogram has a fair degree of accuracy in predicting the survival rates of BC patients.

In data mining, PCA and t-SNE are commonly utilized. In both the training (4(a) and 4(b)) and test sets, we discovered that risk models can effectively discriminate risk patients (Figures 4(c) and 4(d)). Between the survival data and the risk model, univariate and multivariate Cox regression analyses were performed. The forest plot revealed that separate survival time parameters in the training (Figures 4(e) and 4(g)) and test sets influenced the risk model (Figures 4(f) and 4(h)). Furthermore, we analysed the DEGs involved in pyroptosis using KEGG pathway enrichment analysis and GO analysis. The enriched biological process (BP) term was linked to the humoral immune response, the enriched molecular function (MF) term to T-cell activation (Figure 4(i)), and the enriched KEGG pathways to the NF kappa B signaling network and T-cell receptor signaling pathway (Figure 4(j)). Risk signatures may be applied intuitively and successfully with nomograms, and outcomes can be predicted with ease. Our nomogram, as shown in Figure 5, has a fair degree of accuracy in predicting the survival rates of BC patients.

### 3.4. Analysis of Immune Scores among Molecular Subtypes

**Figure 6:**
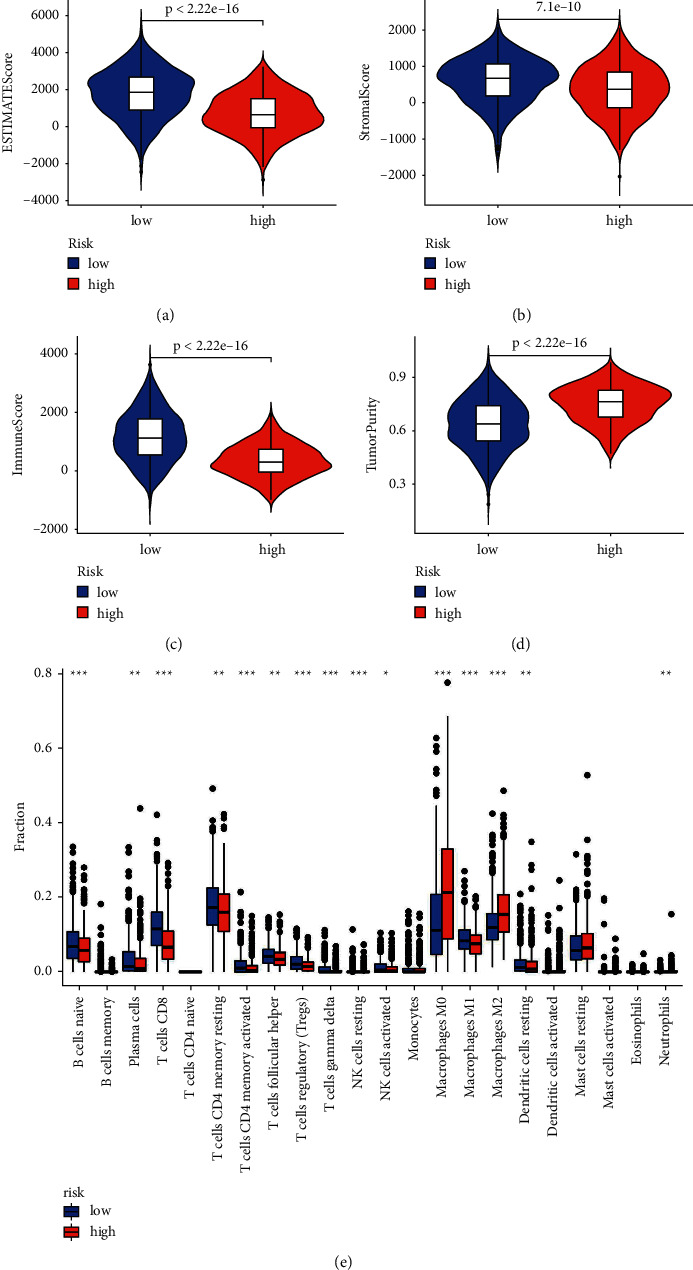
Immune scores among molecular subtypes. Our model can distinguish the (a) estimate score, (b) stromal score, (c) immune score, and (d) purity of tumours well and (e) has a good predictive effect in evaluating 28 types of immune cells.

**Figure 7:**
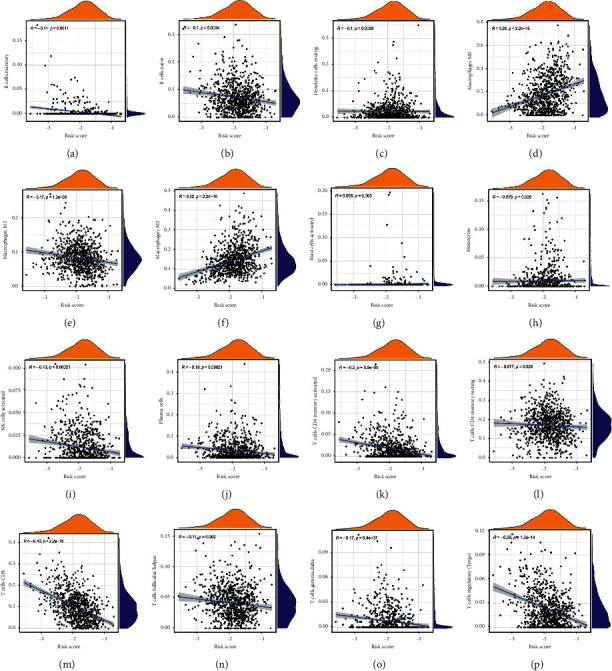
Illustrations of 28 different types of immunological cells. The signature has a good predictive effect in immune cells, including (a). B-cell memory, (b). B naive cells, (c). dendritic cells resting, (d). macrophages M0, (e). macrophages M1, (f). macrophages M2, (g). mast cells activated, (h). monocytes, (i). NK cells activated, (j). plasma cells, (k). T-cell CD4 memory activated, (l). T-cell CD4 memory resting, (m). T-cell CD8, (n). T follicular helper cells, (o). T-cell gamma delta, and (p). regulatory cells (Tregs).

**Figure 8:**
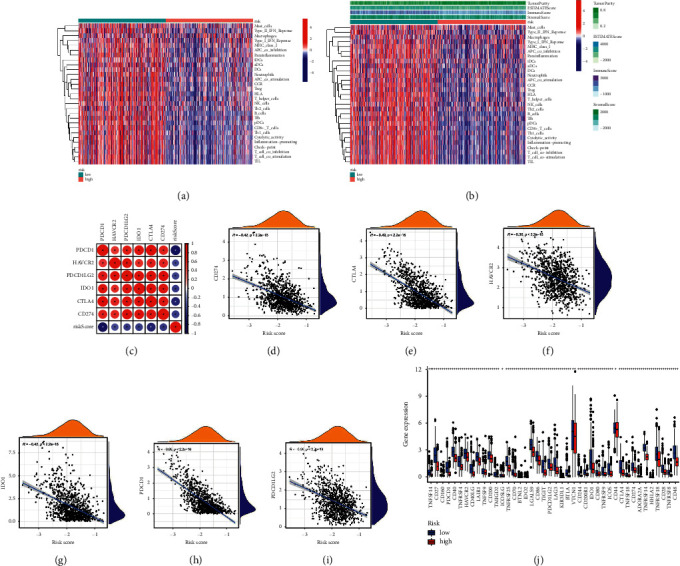
The pyroptosis-related signature has a good evaluation effect on the tumour microenvironment score and checkpoint therapy. The heatmap was used for evaluation (a). The tumour microenvironment score and (b). Immune cells. (c-j). The pyroptosis-related signature has a good evaluative effect in a checkpoint therapy.

We used ESTIMATE software, which can predict the tumour microenvironment score. Our model can distinguish the estimate score (Figure 6(a)), stromal score (Figure 6(b)), immune score (Figure 6(c)) and purity of the tumour (Figure 6(d)) well. The pyroptosis-related signature has a good predictive effect in evaluating 28 types of immune cells (Figure 6(e)), including B cells memory (Figure 7(a)), B cells naive (Figure 7(b)), dendritic cells resting (Figure 7(c)), macrophages M0 (Figure 7(d)), macrophages M1 (Figure 7(e)), macrophages M2 (Figure 7(f)), mast cells activated (Figure 7(g)), monocytes (Figure 7(h)), NK cells activated (Figure 7(i)), plasma cells (Figure 7(j)), T cells CD4 memory activated (Figure 7(k)), T cells CD4 memory activated resting (Figure 7(l)), T cells CD8 (Figure 7(m)), T cells follicular helper (Figure 7(n)), T cells gamma delta (Figure 7(o)), and T cells regulatory (Tregs) (Figure 7(p)). Furthermore, a heatmap was used to evaluate the tumour microenvironment score (Figure 8(a)) and immune cells (Figure 8(b)). Patients with lower risk scores had a better response to ICI therapy, indicating that the pyroptosis-related signature had well evaluation effect in checkpoint therapy (Figures 8(c)–8(j)).

### 3.5. The Role of APOBEC3D, TNFRSF14, and RAC2 in BC

**Figure 9:**
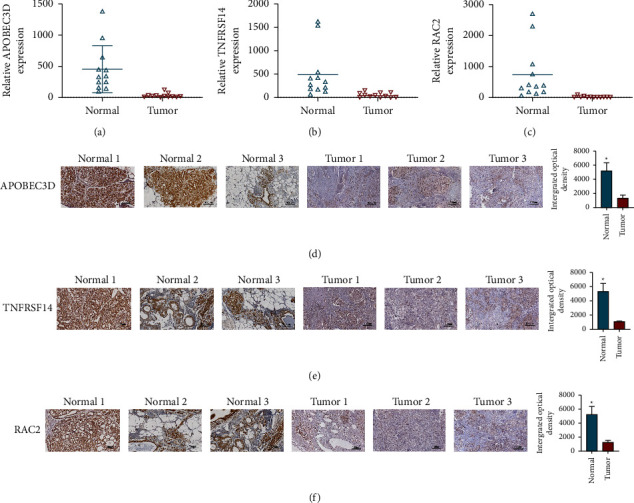
The expression of APOBEC3D, TNFRSF14, and RAC2 in BC tissues. In BC tissues, APOBEC3D, TNFRSF14, and RAC2 were expressed at lower levels in the high-risk group, according to results of (a-c) qPCR and (d-f) immunohistochemistry.

**Figure 10:**
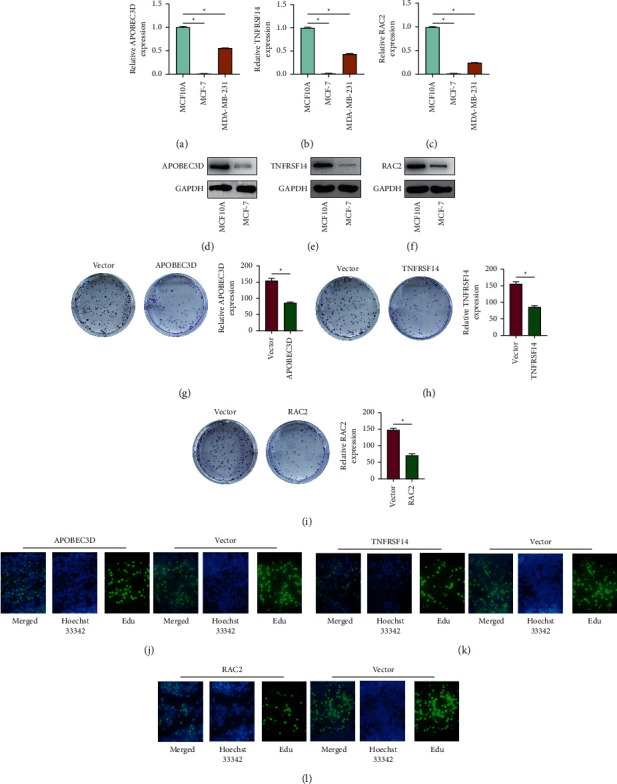
The biological functions of APOBEC3D, TNFRSF14, and RAC2 in BC cells. (a-c) PCR results showed that the model gene expression was lower in MCF-7 and MDA-MB-231 cells. (d-f) Western blotting experiments showed that the APOBEC3D, TNFRSF14, and RAC2 protein expression were weaker in MCF-7 cells. Results of colony formation (g-i) and EdU (j-l) assays revealed that overexpression of APOBEC3D, TNFRSF14, and RAC2 greatly suppressed MCF-7 cell proliferation.

Based on the pyroptosis-related signature gene (APOBEC3D, TNFRSF14, and RAC2) risk score, the expression of the genes was investigated using qPCR and immunohistochemistry. The findings of the qPCR (Figures 9(a)–9(c)) and immunohistochemistry (Figures 9(d)–9(f)) analyses revealed that the APOBEC3D, TNFRSF14, and RAC2 expressions in BC tissues. We examined the expression of APOBEC3D, TNFRSF14, and RAC2 in BC cells, and the PCR results showed that the APOBEC3D, TNFRSF14, and RAC2 expressions were significantly low in MCF-7 and MDA-MB-231 cells (Figures 10(a)–10(c)). Since APOBEC3D, TNFRSF14, and RAC2 were all minimally expressed in MCF-7 cells, we next selected it for further study. Western blotting experiments showed that the protein expression of APOBEC3D, TNFRSF14, and RAC2 were weaker in MCF-7 cells (Figures 10(d)–10(f)). Furthermore, the biological functions of APOBEC3D, TNFRSF14, and RAC2 were investigated. To test the proliferation of MCF-7 cells, we used an overexpression method for APOBEC3D, TNFRSF14, and RAC2. The proliferation ability of MCF-7 cells was determined using colony formation (Figures 10(g)–10(i)) and EdU assays (Figures 10(j)–10(l)). The results revealed that overexpression of APOBEC3D, TNFRSF14, and RAC2 greatly suppressed MCF-7 cell proliferation.

## 4. Discussion

Breast cancer (BC) is the most common women malignant tumor all over the world [13], with significant heterogeneity and molecular features [14]. A growing number of studies have found that pyroptosis is important in the course of cancer and has a link to the effects of some chemotherapy medications [15]. Furthermore, numerous studies have revealed that pyroptosis-related genes may be potential therapeutic targets and have a link to breast cancer chemotherapy drugs [16–18]. However, only a few pyroptosis-related markers have been discovered, which are intimately linked to the prognosis and immune infiltration of BC.

In the present study, we identified pyroptosis-related genes from TCGA-BC, which used them to construct a novel predictive molecular model for BC (APOBEC3D, TNFRSF14, and RAC2). For the risk analysis, we used the training set (TCGA-BC dataset) and test set (GSE37751 dataset) to determine whether our signature was feasible. Our signature had good classification efficiency of the Kaplan–Meier survival curves, ROC curves, and risk score distributions for OS prediction. Furthermore, we compared our model with other risk models and our model has a more effective result. The risk model is the influence of survival time and accuracy for forecasting the survival rates of BC patients in Cox regression analysis and the nomogram. Our model can identify the estimate score, stromal score, immunological score, and purity of tumors well, which is another key point of the risk model. Additionally, the pyroptosis-related signature has a good predictive effect in evaluating immune cells and checkpoint therapy. The qPCR and immunohistochemistry results showed that APOBEC3D, TNFRSF14, and RAC2 were expressed at lower levels in stages III and IV (high-risk group) in BC tissues. Furthermore, the results of biological functions revealed that overexpression of APOBEC3D, TNFRSF14, and RAC2 greatly suppressed MCF-7-cell proliferation. The findings of the current study provide more effective tools for predicting prognosis and immune infiltration in BC, and the signature genes may serve as potential targets for BC, which have not been found in previous studies.

Here, pyroptosis-related APOBEC3D, TNFRSF14, and RAC2 genes were considered risk genes for BC. It has been reported that the expression of APOBEC3D [19, 20], TNFRSF14 [21, 22], and RAC2 [23, 24] is dysregulated and is a potential target for therapy in cancer. In BC, TNFRSF14 and RAC2 are prognostic markers, which is consistent with our findings. For APOBEC3D, we report for the first time that APOBEC3D could be used as a new molecular marker in BC. However, our study also has some limitations: 1. A small number of clinical samples were used to test the pyroptosis-related APOBEC3D, TNFRSF14, and RAC2 genes. In future studies, we will expand the number of samples for research. 2. The function of APOBEC3D *in vitro* experiments will be analysed in the future.

Finally, our research study presents a unique pyroptosis-related genes prognostic molecular model (APOBEC3D, TNFRSF14, and RAC2) that could be used to assess prognostic risk and immune infiltration in BC. The overexpression of APOBEC3D, TNFRSF14, and RAC2 significantly reduced MCF-7 cell proliferation, according to the results of biological functions. Our pyroptosis-related signature could be utilized to assess prognosis and immune infiltration and it could be used to identify potential targets for BC.

## Data Availability

The datasets used or analysed during the current study are available from the corresponding author on reasonable request.
